# Variation in antibiotic treatment for diabetic patients with serious foot infections: A retrospective observational study

**DOI:** 10.1186/1472-6963-10-193

**Published:** 2010-07-06

**Authors:** Benjamin G Fincke, Donald R Miller, Cindy L Christiansen, Robin S Turpin

**Affiliations:** 1 Center for Health Quality Outcomes and Economic Research (CHQOER), Edith Nourse Rogers Memorial Veterans Hospital, 200 Springs Road, Bedford, MA 01730, USA; 2 Boston University School of Public Health, 715 Albany Street, Boston, MA 02118, USA; 3 Baxter International, Baxter Healthcare DF5-3W, One Baxter Parkway, Deerfield, IL 60015, USA; 4 Department of Health Policy, Jefferson Medical College, Philadelphia, PA, USA

## Abstract

**Background:**

Diabetic foot infections are common, serious, and diverse. There is uncertainty about optimal antibiotic treatment, and probably substantial variation in practice. Our aim was to document whether this is the case: A finding that would raise questions about the comparative cost-effectiveness of different regimens and also open the possibility of examining costs and outcomes to determine which should be preferred.

**Methods:**

We used the Veterans Health Administration (VA) Diabetes Epidemiology Cohorts (DEpiC) database to conduct a retrospective observational study of hospitalized patients with diabetic foot infections. DEpiC contains computerized VA and Medicare patient-level data for VA patients with diabetes since 1998, including demographics, ICD-9-CM diagnostic codes, antibiotics prescribed, and VA facility. We identified all patients with ICD-9-CM codes for cellulitis/abscess of the foot and then sub-grouped them according to whether they had cellulitis/abscess plus codes for gangrene, osteomyelitis, skin ulcer, or none of these. For each facility, we determined: 1) The proportion of patients treated with an antibiotic and the initial route of administration; 2) The first antibiotic regimen prescribed for each patient, defined as treatment with the same antibiotic, or combination of antibiotics, for at least 5 continuous days; and 3) The antibacterial spectrum of the first regimen.

**Results:**

We identified 3,792 patients with cellulitis/abscess of the foot either alone (16.4%), or with ulcer (32.6%), osteomyelitis (19.0%) or gangrene (32.0%). Antibiotics were prescribed for 98.9%. At least 5 continuous days of treatment with an unchanged regimen of one or more antibiotics was prescribed for 59.3%. The means and (ranges) across facilities of the three most common regimens were: 16.4%, (22.8%); 15.7%, (36.1%); and 10.8%, (50.5%). The range of variation across facilities proved substantially greater than that across the different categories of foot infection. We found similar variation in the spectrum of the antibiotic regimen.

**Conclusions:**

The large variations in regimen appear to reflect differences in facility practice styles rather than case mix. It is unlikely that all regimens are equally cost-effective. Our methods make possible evaluation of many regimens across many facilities, and can be applied in further studies to determine which antibiotic regimens should be preferred.

## Background

Foot infections in diabetic patients are among the most frequent precipitating causes of hospitalization and amputation[1-3]. They may result from many different microbial species, either singly or in combination, and response to treatment may be poor, particularly when vascular disease is also present, as is common in diabetes. The Infectious Diseases Society of America has issued a guideline in an effort to standardize and improve care[4,5]. Uncertainty remains, however, regarding the optimal antibiotic regimens for the various types of infection that may occur[6-8]. Lack of consensus about optimal treatment means that there is substantial latitude in the choice of antibiotics for diabetic foot infections, with the likely result that there will be substantial variation in practice. The aim of this study was to determine whether this is the case. To the degree that such variation can be documented, it would raise questions about the comparative cost-effectiveness of different regimens and also open the possibility of examining costs and outcomes in order to determine which regimens should be preferred.

The Veterans Health Administration is the largest integrated health care system in the country and includes 147 hospitals. It has an excellent computerized patient record system, which contains patient demographics, diagnoses, diagnostic studies, treatments received and patient outcomes. With this information it is possible to examine the antibiotic regimens of large numbers of diabetic patients with foot infections and the degree to which practice patterns vary across facilities.

In previous work, we reported the construction of a classification system for diabetic foot infections that is expressly designed for use with computerized medical data. We applied it to a database that contains all diabetic patients in the Veterans Health Administration (VA), and found support for our classification in its correlation with patient characteristics, treatments received, and outcomes, including rehospitalization, amputation, transition to long-term care, and death [9].

In the present study, we have used our classification system to identify diabetic patients with serious active foot infections, as defined by hospitalization with isolated cellulitis or cellulitis accompanied by gangrene, osteomyelitis, or ulcer. We have also developed and applied methods for assessing variation in complex multi-drug antibiotic regimens to examine how the treatment of these patients varies across hospitals in the VA healthcare system. We have found that there is large variation in treatment across facilities and that there is more variation across facilities than across different types of infection. This suggests that practice styles play a larger role in the choice of regimen than does case mix.

## Methods

### The Study Population and Source Data

This study was approved by the Institutional Review Board (IRB) of the Edith Nourse Rogers VA Medical Center in Bedford, Massachusetts. It was conducted in the population of hospitalized veteran diabetic patients receiving care from the Veterans Health Administration (VA) in fiscal year (FY) 2006. We used the national VA Diabetes Epidemiology Cohorts (DEpiC), a linked, computerized research database that serves as a registry of virtually all VA patients with diabetes. Patients are included in DepiC on the basis of having two of the following ICD-9CM codes within a 24-month period: 250.xx, 357.2, 362.0, or 366.41. It contains patient-level data on medical visits, pharmacy and laboratory data, with diagnoses and procedures for VA and non-VA care (from Medicare claims data)[10,11]. For this study, we used ICD-9CM diagnosis and procedure codes from VA inpatient files along with inpatient antibiotic prescriptions. The records for inpatient antibiotics include generic name of the medication and dose, with separate entries for each date that the antibiotic was administered. For this analysis, we did not include antibiotics prescribed on an outpatient basis or any non-VA hospitalizations.

### Identification and classification of diabetic foot infections

**Figure 1:**
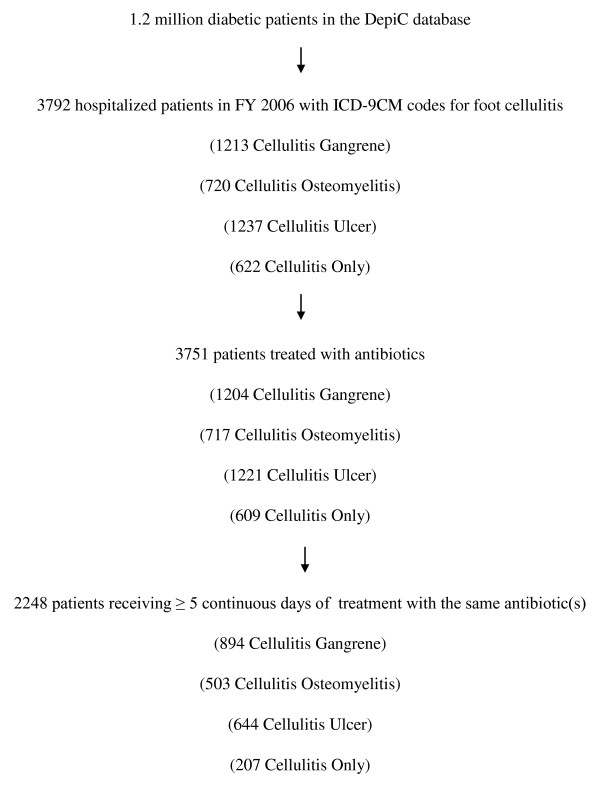
**Study population**.

Our methods for the identification and classification of diabetic foot infections have been described elsewhere[9]. In brief, we used ICD-9-CM codes to identify diabetic patients who had any of the various codes that indicate foot infection during a VA hospitalization in FY2006. Some patients proved to have coexisting foot infections of different types. We reasoned that the most severe of these infections would determine the strength of association with such things as response to treatment, length of hospital stay, amputation rate, and other outcomes. Therefore, we ranked the infections in a presumptive order of severity and assigned the infection to the most severe category for which they had an ICD-9-CM code. Our presumptive order was Gangrene > Osteomyelitis > Foot ulcer > Cellulitis/abscess of foot > Cellulitis/abscess of toe > Paronychia. In the present study, we first identified all patients with ICD-9-CM codes for cellulitis/abscess of the foot and then sub-grouped them according to whether they had cellulitis/abscess plus codes for gangrene, osteomyelitis, skin ulcer, or none of these. If cellulitis/abscess was accompanied by codes for more than one of the foregoing 3 conditions, then we chose the one that was more severe. Figure 1 is a flow diagram that shows the genesis of the study population. The ICD-9-CM codes that we used are given in the appendix.

We assumed that patients were under treatment for diabetic foot infection while in the hospital if, during that hospitalization, they were assigned an ICD-9-CM code for cellulitis of the foot.

### Antibiotic treatment of patients with diabetic foot infections

First, we eliminated from consideration antibiotics that would not be used in the treatment of diabetic foot infections. These included antivirals, antifungals, and antiparasitics, as well as select antibacterial agents, including antituberculosis agents other than rifampin, agents used only for urinary tract infections (e.g. nitrofurantoin, methenamine), and antibacterial agents with a very narrow spectrum of activity, such as penicillin G and spectinomycin. We then evaluated antibiotic prescribing in the following ways:

1. The proportion of patients treated with an antibiotic and the initial route of administration on the first day that any antibiotic was given.

2. The first antibiotic *regimen *prescribed for each patient, defined as treatment with the same antibiotic, or combination of antibiotics, for at least 5 continuous days. This may not be the same as the antibiotics that were given on the first day. We chose this duration of time to allow for consultation about the choice of antibiotics, integration of microbiological information, and observation of clinical response. Also, duration sufficient to observe clinical response is a necessary requirement for future studies to determine which regimens should be preferred.

3. The antibacterial *spectrum *of the first antibiotic regimen prescribed for each patient. We used the Medical Letter on Drugs and Therapeutics Handbook of Antimicrobial Therapy to determine whether an antibiotic was a recommended agent for: gram-positive organisms other than S. aureus; methicillin sensitive S. aureus; methicillin resistant S. aureus; Ps. aeruginosa; and B. fragilis[12]. (We considered regimens to target B. fragilis only if they contained either clindamycin or metronidazole.) In addition, we divided the gram-negative spectrum against organisms other than Ps. aeruginosa into broad and narrow. We classified ampicillin, amoxicillin, and second generation cephalosporins as narrow spectrum agents and the remaining agents as broad spectrum.

### Statistical analyses

We used a Bayesian model and an adjusted likelihood method[13] to estimate values of true rates by facilities. This method shrinks the estimate toward the overall mean, considers the strength of the information about a hospital's true rate relative to the variation across hospitals, and allows estimates from hospitals with small sample sizes[14].

Estimates of the true mean rates incorporate information from a facility's observed rate with information from the distribution of rates of all facilities. If the information from a facility is weak, i.e., the sample size at the facility is small, the estimate of the true rate will be pulled closely toward the overall rate; when the information is strong, the estimate of the true rate will be closer to the facility's observed rate. Although there is statistical error associated with our estimates of true rates, the Bayesian model lets us focus on the parameters of interest rather than focusing on observed rates where random variation is expected.

We characterized the variation in true rates in three ways: range, interquartile range, and range/median. The range is the difference between the lowest and highest values. The interquartile range is the difference between the lowest and highest values in the middle two quartiles of the data. The range/median is similar to the coefficient of variation (standard deviation divided by the mean) in that large values mean that the range of true rates is large relative to the typical value of the true rates. A large value could indicate one or more extreme outliers or true rates with large variation but without extreme outliers.

## Results

### Study population

There were nearly 1.2 million veteran VA patients with diabetes in DEpiC in FY 2006. We identified 3,792 who had a VA hospitalization in that year with ICD-9-CM codes classifiable into one of our four categories of cellulitis. Of these, 3751 (98.9%) were treated with one or more antibiotics and 2248 (59.3%) received at least 5 continuous days with the same antibiotic(s). Table 1 shows the characteristics of our patient population overall and of the subpopulations of patients with Cellulitis-Gangrene, Cellulitis-Osteomyelitis, Cellulitis-Ulcer, and Cellulitis-Only. Overall, more than 98% were men, the average age was 65, and 63% were white. There was some variation in these characteristics across the different categories of cellulitis. Patients with gangrene were slightly older (average age 67) and there were fewer white patients among those with osteomyelitis (54.4%).

**Table 1:** Demographics of patients with different categories of cellulitis

	Total		Cellulitis and Gangrene		Cellulitis and Osteomyelitis		Cellulitis and Ulcer		Cellulitis Only	
	**N**	**%**	**N**	**%**	**N**	**%**	**N**	**%**	**N**	**%**

	3,792	100	1,213	32.0	720	19.0	1,237	32.6	622	16.4

Sex										
Male	3,748	98.8	1,203	99.2	713	99.0	1,220	98.6	612	98.4

Age in 2006 Mean (SD)	65	(10.6)	67	(10.3)	63	(10.4)	64	(10.5)	64	(11.2)

Race										
White	2,371	62.5	757	62.4	392	54.4	817	66.0	405	65.1
African American	440	11.6	168	13.8	84	11.7	119	9.6	69	11.1
Other	245	6.5	94	7.8	56	7.8	60	4.9	35	5.6
Unknown	736	19.4	194	16.0	188	26.1	241	19.5	113	18.2

### Proportion of patients treated with antibiotics and routes of administration

**Figure 2:**
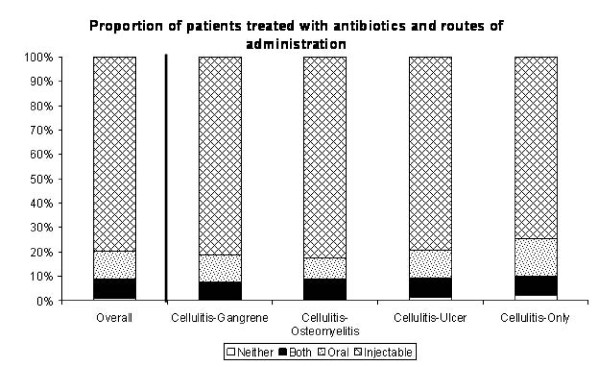
**Proportion of patients receiving antibiotics and routes of administration**.

In this analysis we included all the antibiotics that were prescribed on the first day that any antibiotic was given. Of 3,792 patients overall who had cellulitis, 3,751 (98.9%) were treated, 79.8% parenterally, 11.5% orally, 7.6% both parenterally and orally, and 1.1% neither (i.e. no antibiotic). The greatest difference in initial route of administration was in the Cellulitis-Only group, in which purely parenteral antibiotics were used least often (74.6%), and purely oral antibiotics most often (15.4%), compared to the other groups (Figure 2).

### First antibiotic regimen prescribed to patients with foot cellulitis

Of the 3,792 total patients with any cellulitis, 1544 (40.7%) never received a course of antibiotics that met our definition of an antibiotic regimen. This reflects the fact that either a single day's missed dose or 1-4 days of an additional antibiotic would disqualify an antibiotic regimen from consideration.

The percentage of patients with no first regimen varied across our four categories of cellulitis. Seventy-four percent of patients with Cellulitis-Gangrene received a first regimen, compared to 70% for Cellulitis-Osteomyelitis, 52% for Cellulitis-Ulcer, and 33% for Cellulitis-Only. Since we know that at least 98% of patients in each group were given antibiotics, these declining percentages reveal a corresponding decrease in the proportion of patients who received an unmodified course of treatment for 5 days or more. A total of 199 different antibiotic regimens were used. This large number reflects the many possible combinations of the 52 different antibiotics that were prescribed.

Table 2 shows the 17 regimens that were used in more than 1% of patients. The columns labeled "Observed Proportion" show the mean percentage of patients receiving each regimen overall, and then separately for each category of cellulitis. In all instances treatment was most often partitioned across the regimens of piperacillin/tazobactam, piperacillin/tazobactam plus vancomycin, and ampicillin/sulbactam.

**Table 2:** Proportion of patients receiving each of the 17 most common first antibiotic regimens

	Overall		Cellulitis and Gangrene		Cellulitis and Osteomyelitis		Cellulitis and Ulcer		Cellulitis Only	
	
	(n = 2248)		(n = 894)		(n = 503)		(n = 644)		(n = 207)	
	
	Observed Proportion (Range*) %									
Piperacillin-tazobactam	16.4	(22.8)	20.1	(23.9)	14.7	(24.2)	14.9	(14.2)	8.7	(11.5)

Piperacillin-tazobactam, Vancomycin	15.7	(36.1)	16.1	(33.9)	18.1	(58.3)	15.4	(21.8)	9.7	(11.3)

Ampicillin-sulbactam	10.8	(50.5)	11.3	(42.5)	10.7	(48.8)	9.5	(30.7)	12.6	(21.4)

Vancomycin	6.9	(13.5)	6.2	(6.1)	9.1	(21.1)	5.7	(7.0)	7.7	(13.8)

Cefazolin	4.2	(12.6)	3.4	(9.2)	3.2	(19.7)	5.6	(14.4)	6.3	(12.1)

Amoxicillin-clavulanate^§^	2.0	(3.3)	1.5	(4.1)	0.6	(10.9)	3.7	(7.3)	2.4	( - )

Ticarcillin-clavulanate	2.0	(48.6)	1.9	(41.6)	1.6	(40.6)	2.5	(43.1)	1.9	( - )

Cephalexin^§^	1.6	(7.3)	0.8	(4.6)	0.8	(26.2)	2.2	(10.1)	4.8	(12.7)

Ertapenem	1.4	(14.9)	1.0	(11.5)	2.6	(19.9)	0.5	(12.1)	1.0	( - )

Ciprofloxacin^§^	1.3	(2.5)	1.2	(6.1)	1.4	(6.1)	1.6	(7.9)	0.5	( - )

Ampicillin-sulbactam, vancomycin	1.2	(3.0)	1.2	(2.7)	1.4	(17.4)	1.1	(3.7)	1.0	( - )

Imipenem	1.2	(4.3)	2.1	(11.5)	0.8	(24.9)	0.3	( - )	1.0	( - )

Ceftriaxone	1.2	(3.8)	0.8	(3.7)	1.4	(6.2)	1.6	(6.9)	1.0	( - )

Ciprofloxacin, clindamycin	1.1	(12.7)	1.5	(21.1)	0.6	(15.6)	0.8	(2.9)	1.4	( - )

Clindamycin	1.1	(3.6)	1.1	(7.2)	0.8	(9.7)	0.8	(10.8)	2.4	( - )

Gatifloxacin^§^	1.1	(4.1)	0.4	(8.1)	1.2	(3.9)	1.9	(23.7)	1.0	( - )

Nafcillin	1.1	(2.8)	0.6	(6.5)	1.4	(7.9)	1.1	(25.1)	2.4	( - )

* Statistic uses shrunken hospital rates (see methods)

^§ ^Given orally

( - ) No range

The rows of the table reveal that there are not large differences in the choice of each regimen across the categories of cellulitis. The greatest difference is 11.4% for Piperacillin-tazobactam (high 20.1% for Cellulitis-Gangrene, low 8.7% for Cellulitis-Only). In general, the largest differences in frequency of use across the different categories of cellulitis occur with Cellulitis-Only, in which a substantially smaller proportion of patients received piperacillin-tazobactam and piperacillin-tazobactam plus vancomycin.

The range of use for each regimen across facilities *within *each category of cellulitis is at least equal to, and usually substantially greater than, the range of observed proportions *across *the different types of cellulitis (i.e. the range of variation across facilities is generally greater than that across the different categories of cellulitis).

**Figure 3:**
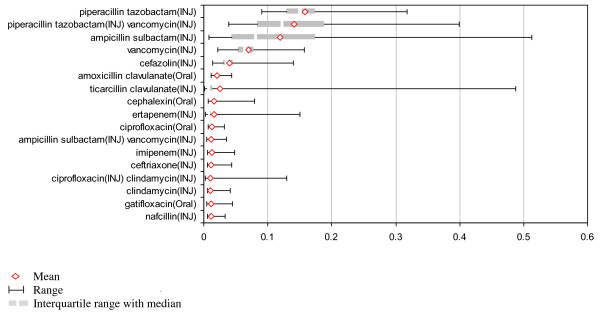
**Proportion of patients receiving each of the 17 most common first antibiotic regimens**.

Figure 3 is a graphical representation of the first column of Table 2, and displays the findings for the study population as a whole. It shows the interquartile range in addition to the full range. The large difference between these indicates that divergent prescribing patterns in a small number of facilities accounts for a large part of the full range of variation that we have observed. Calculation of the range to median ratio, an additional measure of variation (data not shown), highlights the antibiotic regimens for which variation in use is particularly great in relation to the median. It specifically identifies ticarcillin/clavulanate, ertapenem, and ciprofloxacin/clindamycin as highly variable. Though the latter two are given to less than 2% of patients overall, the range of usage is very large by comparison to the median.

### Antibacterial spectrum of first antibiotic regimen prescribed to patients with foot infections

In this analysis we collapsed the extensive array of antibiotic regimens into smaller groups on the basis of their antibacterial spectra. If more than one regimen was used, we assigned the spectrum based on the first. Only 10 different spectra were used in more than 1.6% of patients. Table 3 shows how these varied across the categories of cellulitis as well as across facilities. The three most frequently used spectra account for 63.7% of treated patients. Their frequency of use corresponds to the breadth of their antibacterial spectrum, with the broadest spectrum being most commonly chosen overall. The hierarchy of use for the three most common spectra is maintained within each type of cellulitis with two exceptions (Cellulitis Gangrene and Cellulitis Only), in both of which the deviation reflects a difference less than two percentage points in the Observed Proportion column. The table shows that there is substantial variation in the breadth of the spectrum that is chosen. The patterns and magnitude of variation are similar to those described for Table 2.

**Table 3:** Proportion of patients receiving each of the 10 most common antibacterial spectra

	Overall		Cellulitis and Gangrene		Cellulitis and Osteomyelitis		Cellulitis and Ulcer		Cellulitis Only	
	
	(n = 2248)		(n = 894)		(n = 503)		(n = 644)		(n = 207)	
	
	Observed Proportion (Range*) %									
G + St(res) G-Ps An	25.8	(33.9)	26.7	(18.3)	26.2	(43.8)	25.8	(11.0)	20.8	(25.1)

G + St G-Ps An	23.2	(22.0)	28.0	(20.3)	20.9	(19.9)	21.0	(15.8)	15.0	(22.0)

G + St G-Br An	14.7	(45.0)	14.3	(37.3)	13.9	(37.0)	15.2	(18.9)	16.4	(28.6)

G + St(res) G-Ps	7.8	(5.5)	6.8	(5.6)	8.2	(10.1)	9.0	(11.0)	7.2	(8.3)

G + St(res)	6.9	(13.8)	6.2	(6.1)	9.5	(20.7)	5.7	(7.0)	7.7	(13.8)

G + St G-	6.1	(11.7)	4.4	(5.4)	4.4	(18.2)	8.1	(13.4)	11.6	(6.2)

G + St	1.9	(2.2)	1.9	(2.9)	2.0	(8.4)	2.2	(2.5)	1.0	( - )

G + St(res) G-Br An	1.9	(1.9)	1.0	(9.6)	2.4	(7.5)	1.9	(8.4)	4.3	(11.3)

G + St An	1.8	(5.1)	1.9	(3.5)	1.0	(15.6)	1.7	(5.2)	3.4	(34.5)

G + St(res) G-Br	1.7	(2.7)	1.1	(5.8)	1.4	(12.4)	2.3	(13.1)	2.9	(18.0)

* Statistic uses shrunken hospital rates (see methods)

( - ) No range

Key: G + St = Methicillin sensitive S. aureus

G + St(res) = Methicillin resistant S. aureus

G- = Narrow spectrum Gram negative

G-Br = Broad spectrum Gram negative

G-Ps = Broad spectrum Gram negative including Ps. aeruginosa

An = Specific anaerobic agents (clindamycin or metronidazole)

## Discussion

There have been numerous studies of variation in antibiotic use in different types of infection [15-22]. The most common underlying premise has been that variations in care will reveal deviations from ideal management, whether judged by medical outcomes, costs, or both. Even when there is not agreement about what constitutes ideal management, there is a presumption that all treatment strategies are unlikely to be equally efficacious, so that the presence of substantial variations reveals the importance of further investigations to determine which approaches are most cost-effective[23,24].

Evaluation of variation in complex multi-drug regimens presents methodological challenges. Perhaps for this reason, most studies of differences in antibiotic treatment have used strategies that simplify the assessment of variation, such as examining any treatment versus no treatment,[18] deviation in treatment from practice guidelines,[17] or differences in antibiotic regimens when treatment is usually with single drugs, as is the case with infections like otitis media and respiratory tract infections[15,16]. We have not found any that have evaluated diabetic patients with foot infection, who are often treated with complex regimens that are not well-suited to the foregoing methods of analysis.

We decided to carry out such a study, first, because diabetic foot infections are such an important cause of hospitalization, amputation, and disability and, second, because there is no generally accepted standard antibiotic regimen, so we expected to find large variations in antibiotic management. We used data derived from the computerized medical records of the largest integrated medical care organization in the country, the Veterans Health Administration. This gave us the power to evaluate variation in a large variety of antibiotic regimens, across a large number of hospitals, in a large number of patients. We have focused on patients where antibiotic choices are most important, namely those with serious foot infections as defined by hospital treatment for cellulitis accompanied by gangrene, osteomyelitis, cutaneous ulcer, or occurring in isolation.

The complex antibiotic regimens often used to treat diabetic foot infections present methodological issues that we needed to address before we could begin. First, we had to take into account that the initial antibiotic prescribed might not necessarily represent a final decision about the preferred antibiotic regimen. To address this we examined what we considered to be a stable "course" of antibiotics, given singly or in combination, without change, over a period of at least 5 days. Such a time frame is a requirement for future studies, which will not be able to evaluate the outcomes of different regimens unless they have been given for long enough to assess clinical response. Second, we recognized that variation in the choice of antibiotics would be governed, in part, by the breadth of the anti-bacterial spectrum desired. Hence, we also examined variation in the antibacterial spectrum of treatment well as variation in the antibiotics themselves.

We have found very large differences in antibiotic use across VA facilities. It is evident in the antibiotics that make up the initial regimen, as well as in the antibacterial spectra of the chosen regimens. The variation may be partly driven by differences in case mix, but this is unlikely, because we did not find substantial differences in case mix across facilities as judged by the comparative prevalence of our different categories of cellulitis. It is probably truly related to foot infections, because the percentage of patients treated with any antibiotic is close to 100%, the great majority of treatments are parenteral, and the spectrum of antibacterial activity is broad, all of which are to be expected in the treatment of these infections[5,25-28]. In addition, there are expected patterns of treatment related to our different categories of cellulitis. In particular, patients with the least complicated infection (Cellulitis-Only) receive more oral treatment and less often receive 5 or more continuous days of the same medication.

There is evidence in this study that antibiotic choice is driven by the existence of differing practice styles across VA facilities. The variation in antibacterial spectrum across facilities within each category of infection almost certainly reflects different views about how broadly to treat. (The difference between the three most commonly used regimens, for example, is whether they cover methicillin resistant S. aureus (MRSA), Ps. aeruginosa, or neither. Each of these organisms is recognized as a cause of serious foot infections, but they are not so common as to mandate routine coverage[29].) There is also evidence for practice variation in the agents chosen to achieve each spectrum, in that the number of spectra is substantially smaller than the number of regimens. Finally, we saw in some facilities a particular preference for one or another of a pair of antibiotics that are very similar in antibacterial spectrum as well as side effects. Facilities almost always used one or the other of these pairs, rarely both. Examples are imipenem versus ertapenem and ticarcillin/clavulanate versus piperacillin/tazobactam.

Even though there is no general agreement about antibiotic choice in the infections we have studied, our findings raise concern about the cost-effectiveness of the care that is being given. It is unlikely that all regimens are equally advantageous. For example, there is some evidence that ampicillin-sulbactam may be more cost-effective than imipenem[30,31]. There is also a controlled trial that has shown ertapenem to be as effective in the treatment of diabetic foot infections as piperacillin/tazobactam (one of the most commonly used agents in our study), and a comparative study of costs estimated ertapenem to be less expensive, primarily because it had to be administered less frequently [32,33]. We note, however, that such studies may not be determinative, because other considerations may be more important. It may be advisable, for example, to hold a broad-spectrum antibiotic in reserve if frequent use is known to generate resistant organisms.

Clearly, further studies are needed to identify which antibiotic regimens are most cost-effective. This study has established a number of methodological approaches that can facilitate the conduct of such investigations using computerized medical data. These include, 1) a classification system for diabetic foot infections that can be applied to computerized medical data; 2) an operational definition of the initial antibiotic regimen, including the requirement to exclude drugs unlikely to be used for foot infection; 3) a taxonomy for classifying the spectra of antibacterial agents that has proven to be useful in evaluating variation in complex antibiotic regimens, and 4) the benefit of using of shrunken means to make possible the analysis of antibiotic variation across large numbers of facilities, each of which may see only a few patients treated with a given antibiotic regimen. These methods set stage for observational studies of the relationship between different antibiotic regimens, treatment outcomes, and costs. They are also applicable to other conditions, such as intra-abdominal infections, that require empiric treatment with broad-spectrum regimens that may be achieved using many different antibiotic combinations.

There are a number of limitations to this study. First, 41% of the patients never met our definition of a stable antibiotic regimen. This occurred more commonly among patients with milder infections, which indicates that our findings regarding the chosen antibiotic regimens are biased toward patients with greater severity. This might result in the use of broader spectrum regimens and therefore reduce observed differences in treatment across our categories of infection. Second, the antibiotic regimens that we studied may have been customized in response to microbiologic data. To explain our results, however, would require very large differences across facilities in the prevalence of organisms such as MRSA and Ps. aeruginosa. In a recent study, each of these was isolated from less than 5% of cultures, which makes this possibility very unlikely[29]. Third, ICD-9CM coding for foot infections is known to contain inaccuracies[34]. Despite this, we have found in prior work that that our classification of foot infections permits meaningful distinctions to be made [9]. Fourth, we do not have information about comorbidities that might influence prescribing, such as allergies or renal disease. While this probably accounts for some variation, it is unlikely that differences across facilities are great enough to explain the large variation in antibiotic use that we observed across hospitals. For example, the most commonly used regimen that would be affected by penicillin allergy (piperacillin-tazobactam) has a range of use across hospitals of 23%, while that of the most common regimen that would be affected by chronic renal disease (piperacillin-tazobactam plus vancomycin) is 36%. Fifth, the great majority of patients in our study were male, but it is unlikely that gender would have a large effect on antibiotic choice. Sixth, there is no gold standard for assigning antibacterial spectra to antibiotic regimens and differences of opinion are likely. Changing the assignment of some of the regimens, however, would not alter our finding that there is substantial variation in antibiotic treatment, it would simply move the source of some of the variation from one spectrum to another. Last, despite the relatively large patient population studied, there were small numbers in many of the facilities and antibiotic groups studied. While we applied Bayesian shrinkage of the means to address this problem, the range of true variation may still have been overestimated.

## Conclusions

We have found large variations in the antibiotic regimens used to treat patients with diabetic foot infections. They appear to reflect facility differences in practice styles rather than case mix. It is unlikely that these regimens are equally cost-effective. The methods we have developed can be applied in further studies to examine both costs and outcomes in order to determine which regimens should be preferred. They also can be extended to the evaluation of any condition, infective or not, that is often treated with a multidrug regimen.

## Competing interests

The authors declare that they have no competing interests.

## Authors' contributions

All authors contributed to the conception, design, interpretation of data and drafting of the manuscript. DMR acquired the data. CLC designed and oversaw the statistical analyses. All authors have read and approved the manuscript

## Appendix - ICD-9CM codes

### Gangrene

040.0 Gas Gangrene

440.24 Atherosclerosis of the extremities with gangrene

785.4 Gangrene but only if any one of the following is also present:

250.7 Diabetes with peripheral circulatory disorders

440.2 Atherosclerosis of native arteries of the extremities

Any condition classifiable to 440.21, 440.22, and 440.23

### Osteomyelitis

730.07 Acute osteomyelitis of ankle and foot

730.17 Chronic osteomyelitis of ankle and foot

730.27 Unspecified osteomyelitis of ankle and foot

730.97 Unspecified infection of bone of ankle and foot

### Ulcer

440.23 Atherosclerosis of the extremities with ulceration

707.14 Ulcer of heel and mid foot

707.15 Ulcer of other part of foot

707.1 Ulcer of lower limbs

### Cellulitis or abscess of foot

680.7 Carbuncle and furuncle of foot, heel, toe

682.7 Cellulitis and abscess of foot, except toes

### Cellulitis or abscess of toe

681.1 Cellulitis and abscess of toe

681.10 Cellulitis, toe nos

### Paronychia

681.11 Onychia and paronychia of toe

## Pre-publication history

The pre-publication history for this paper can be accessed here:

http://www.biomedcentral.com/1472-6963/10/193/prepub
